# Impact of the Consumption of Tea Polyphenols on Early Atherosclerotic Lesion Formation and Intestinal *Bifidobacteria* in High-Fat-Fed ApoE^−/−^ Mice

**DOI:** 10.3389/fnut.2016.00042

**Published:** 2016-12-21

**Authors:** Zhen-Lin Liao, Ben-Hua Zeng, Wei Wang, Gui-Hua Li, Fei Wu, Li Wang, Qing-Ping Zhong, Hong Wei, Xiang Fang

**Affiliations:** ^1^College of Food Science, South China Agricultural University, Guangzhou, China; ^2^Department of Laboratory Animal Science, College of Basic Medical Sciences, Third Military Medical University, Chongqing, China

**Keywords:** atherosclerosis, *Bifidobacteria*, gut microbiome, tea polyphenols, C57BL/6 ApoE^−/−^ mice

## Abstract

There is an increasing interest in the effect of dietary polyphenols on the intestinal microbiota and the possible associations between this effect and the development of some cardiovascular diseases, such as atherosclerosis (AS). However, limited information is available on how these polyphenols affect the gut microbiota and AS development. This study was designed to evaluate the modulation of dietary tea polyphenols (TPs) on intestinal *Bifidobacteria* (IB) and its correlation with AS development in apolipoprotein E-deficient (ApoE^−/−^) mice. Fifty C57BL/6 ApoE^−/−^ mice were randomized into one of the five treatment groups (*n* = 10/group): control group fed normal diet (CK); a group fed a high-fat diet (HFD); and the other three groups fed the same HFD supplemented with TPs in drinking water for 16 weeks. The total cholesterol and low-density lipoprotein cholesterol (LDL-C) were decreased significantly (*P* < 0.05) after TP interference. In addition, the TP diet also decreased the plaque area/lumen area (PA/LA) ratios (*P* < 0.01) in the TP diet group. Interestingly, copies of IB in the gut of ApoE^−/−^ mice were notably increased with TP interference. This increase was dose dependent (*P* < 0.01) and negatively correlated with the PA/LA ratio (*P* < 0.05). We conclude that TPs could promote the proliferation of the IB, which is partially responsible for the reduction of AS plaque induced by HFD.

## Introduction

Atherosclerosis (AS) is a leading cause of death in Western countries, which is a complex pathophenotype governed by genetic and environmental determinants. High-fat diet (HFD) is one of the environmental risk factors for the development of AS, which may cause changes in the intestinal microbial communities and may lead to obesity and low-grade intestinal inflammation (1). Moreover, the gut microbiota itself is an environmental risk factor for the development of AS (2).

Intestinal microbial communities may influence the efficiency of dietary energy uptake and consequently influence susceptibility to obesity. A novel pathway linking dietary lipid intake, intestinal microbiota, and AS was identified, and the regulation of surface expression levels of macrophage scavenger receptors is well known to participate in the atherosclerotic process (3). Gut microbiota is also believed to be responsible for the control of metabolic endotoxemia, low-grade inflammation, and obesity (4). Additionally, a strong correlation has been observed between microbiota composition and AS development (5, 6). Infection of *Helicobacter cinaedi* was found to alter expression of cholesterol receptors and transporters in cultured macrophages and caused foam cell formation, and enhanced AS in hyperlipidemic mice (7).

The principal component of AS plaques is lipids, which are derived from plasma lipids such as total cholesterol (TC), triglycerides (TG), and low-density lipoprotein cholesterol (LDL-C). High-density lipoprotein cholesterol (HDL-C) and LDL-C in serum are the major factors affecting the development of AS lesions (8). The concentration of serum LDL-C is positively correlated with the incidence of AS (9). Serum TG may also promote plaque formation (10, 11), which further promotes AS (12). *Bifidobacterium* is one of the major genera of *Actinobacteria* that make up the colon microflora in mammals. Phylum *Actinobacteria* was considered to suppress inflammation and obesity (13, 14). Moreover, *Bifidobacteria* may produce short-chain fatty acids to decrease gut pH (15), form a biological barrier, and secret antimicrobial compounds to attenuate harmful bacteria (16, 17). Populations of *Bifidobacterium* spp. are negatively correlated with serum TC and non-HDL-cholesterol level, autoantibodies against oxidized LDL, and AS lesion size (5). However, serum HDL-C level is positively correlated with *Bifidobacterium* spp. populations (5). Therefore, the diet pattern that modulates the intestinal *Bifidobacteria* (IB) may further influence the development of AS.

Tea polyphenols (TPs) contain catechins, flavonoids and flavonols, anthocyanins, and phenolic acids. They were identified to significantly reduce AS plaque area (18, 19) and have effects against coronary heart disease, high blood cholesterol concentrations, and high blood pressure (20). Loke et al. hypothesized that polyphenols in foods can slow down the inflammatory response, which reduces NO bioavailability and therefore help ApoE^−/−^ mice fight against AS (1). TPs may adjust the composition of the gut microbiome to maintain intestinal micro-ecological balance (21, 22), and protect and promote the development of probiotics (23, 24). This phenomenon has also been observed in other food-derived polyphenols (25).

We hypothesize that TPs may modulate fat metabolism and AS development by adjusting the gut *Bifidobacteria* in some degree. The present study was designed to understand the role of TPs on gut *Bifidobacteria*, lipid metabolism, and AS by using different dosages of TPs fed to ApoE^−/−^ mice. The relationship among dietary TPs, gut *Bifidobacteria*, and AS was also explored.

## Materials and Methods

### Animals, Diet, and Sample Collection

Fifty 8-week-old-specific pathogen-free (SPF) C57BL/6 ApoE^−/−^ mice were randomized into one of the five treatment groups (*n* = 10/group, each half male and female): (1) high-dose TPs group (TPH), with 1.6 g/L of TP in the drinking water; (2) middle-dose TP group (TPM), with 0.8 g/L of TP in the drinking water; (3) low-dose TP group (TPL), with 0.4 g/L of TP in the drinking water; (4) high-fat control group (HFD); and (5) normal diet group (CK). In each group, 30 g/L sucrose was added to the drinking water to mask the bitterness of TP (19). The CK group was fed with regular feed (formulation is shown in Table S1 in Supplementary Material); the remaining four groups were fed a high-fat high-cholesterol feed (regular feed 83.25 g, fat 15 g, cholesterol 1.25 g, and cholic acid 0.5 g). The mice were housed in cages of the Third Military Medical University, Department of Experimental Animal Center of SPF animal house, at 23 ± 2°C with relative air humidity of 50 ± 5% on a 12-h light–dark cycle. All feeds were sterilized by ^60^Co gamma radiation (42.5 millirad, Radiation Centre of Third Military Medical University).

The study was approved and supervised by the Ethics Committee of the Third Military Medical University Chongqing, China. SPF C57BL/6 ApoE^−/−^ mice (No. SCXK 2012-0003; SYXK 2012-0002) used in this study were provided by the Department of Laboratory Animal Science of the Third Military Medical University.

Drinking water was replaced daily according to the formulation, and food consumption and body weight changes were recorded weekly. Two to five feces pellets from each cage were weekly collected stored in micro-tubes at −80°C until use.

Tea polyphenols were provided by Jiangxi Lvkang Natural Products Co., Ltd., Yichun, China (main components are shown in Table S2 in Supplementary Material).

### DNA Extraction

Approximately 20 mg of mice fecal sample was enclosed in a pre-aseptic 2-mL screw-cap tube (Axygen, Union City, CA, USA) with 0.1-g zirconia beads (diameter of 0.1 mm, BioSpec, Bartlesville, OK, USA), 250 μL lysis buffer (0.5M NaCl, 50 mM EDTA, 50 mM Tris–HCl pH 8.0, and 4% SDS), and 100 μL phenol:chloroform:isoamyl alcohol (25:24:1) (v/v). Specimens were homogenized with a MiniBeadBeater-16 (BioSpec) for 2 min at maximum speed, and then 1–5 μL of DNA-free RNAase (10 mg/μL) was added to remove residual RNA. The DNA was purified with a phenol–chloroform extraction. The DNA concentration was measured with a NanoDrop ND-1000 spectrophotometer (Thermo, Wilmington, DE, USA) at 260 and 280 nm, and DNA integrity was examined by electrophoresis in 0.8% (w/v) agarose gels (26).

### ApoE^−/−^ Mice Abdominal Aorta Section and Atherosclerosis Plaque Analysis

After treatment for 16 weeks, ApoE^−/−^ mice were anesthetized, their limbs were fixed on an anatomic plate, and their chest and abdomen were disinfected with 75% ethanol. After the thoracic cavity was opened to expose the heart, the left ventricle and right atrial appendage were cut. A perfusion needle was immediately inserted into the left ventricle after the cut to perfuse saline and 10% formaldehyde into heart for 40 min. About 10-mm specimens were cut from the abdominal aorta, fixed with 10% formalin, and embedded in paraffin after 48 h. Each aorta sample was stained with hematoxylin–eosin (HE) (27). The lesion area was measured by means of direct image capture from optical microscopy (Nikon digital system, Tokyo, Japan) and quantified using Image Pro Plus 6.0 (Media Cybernetics, Warrendale, PA, USA) software.

### Serum Lipid and Lipoprotein Analyses

After 16 weeks, 3ApoE^−/−^ mice were randomly selected from each group and anesthetized with 0.5% barbital sodium solution, and 0.2-mL whole blood samples from each mouse were drawn and stored in sterilized Eppendorf tubes. The blood was allowed to clot at room temperature before the serum was separated by centrifugation at 1000 *g* for 10 min. Serum was transferred to sterilized Eppendorf tubes and stored at −80°C until use. Serum parameters were determined by an Olympus Biochemical Auto-analyzer (Olympus AU 2700 Auto-analyzer, Olympus Corporation, Tokyo, Japan) using reagents from Olympus Diagnostics (28).

### Quantitation of *Bifidobacteria*

A *Bifidobacteria* genus quantitative PCR standard curve was established by cloning a standard *Bifidobacterium* strain *Bifidobacterium breve* LMG11042 16S rRNA gene into a pMD19-T vector, as Bartosch et al. previously described (29). Plasmid standards and samples were assayed simultaneously in triplicate.

### The qRT-PCR Analysis of *Bifidobacterium*

16S rRNA genes was extracted by using SYBR PCR reagents; the extracted genes were then analyzed by an iCycleriQ real-time detection system (Bio-Rad, Hercules, CA, USA) compatible with the iCycler Optical System Interface software program (Bio-Rad) and *Bifidobacterium* primers (Bifid-real F, 5′-GCGTGCTTAACACATGCAAGTC-3′; Bifid-real R, 5′-CACCCGTTTCCAGGAGCTATT-3′) (30). The 20-μL reaction mixture contained 2-μL DNA (diluted plasmid standards or samples), 0.2 μM of each primer, and appropriate amounts of other reagents as recommended by the manufacturer. The PCR thermocycling was programed to run the first cycle at 95°C for 3 min, followed by 35 cycles of denaturation at 95°C for 30 s, and annealing/elongation at 60°C for 1 min. After amplification, melting curve analysis of the PCR products was performed from 60 to 94°C, with increments of 0.5°C per 10 s. The copy number of the target sequence was calculated as previously described (31).

### Statistical Analysis

Statistical analysis was performed using GraphPad Prism Software for Windows OS (version 6.05, Graphpad Software, La Jolla, CA, USA). Statistically significant differences between serum TC, TG, HDL-C, and LDL-C content and AS plaque area between the five treatment groups were determined by the non-parametric one-way ANOVA test with default parameters all the time. The Tukey’s multiple comparisons test was used for multiple pairwise comparisons and a dose–response relationship. A dose–response meta-analysis was performed to examine a potential non-linear relationship between TP dose, AS plaque area, and LgN (copies of *Bifidobacteria*), and a dose–response meta-analysis was conducted using generalized least squares regression test with EViews Software for Windows (version 8.0, IHS Global GNC, PA, USA). *P* < 0.05 was considered statistically significant.

## Results

### Body Weight and Feed Consumption of ApoE^−/−^Mice

**Figure 1:**
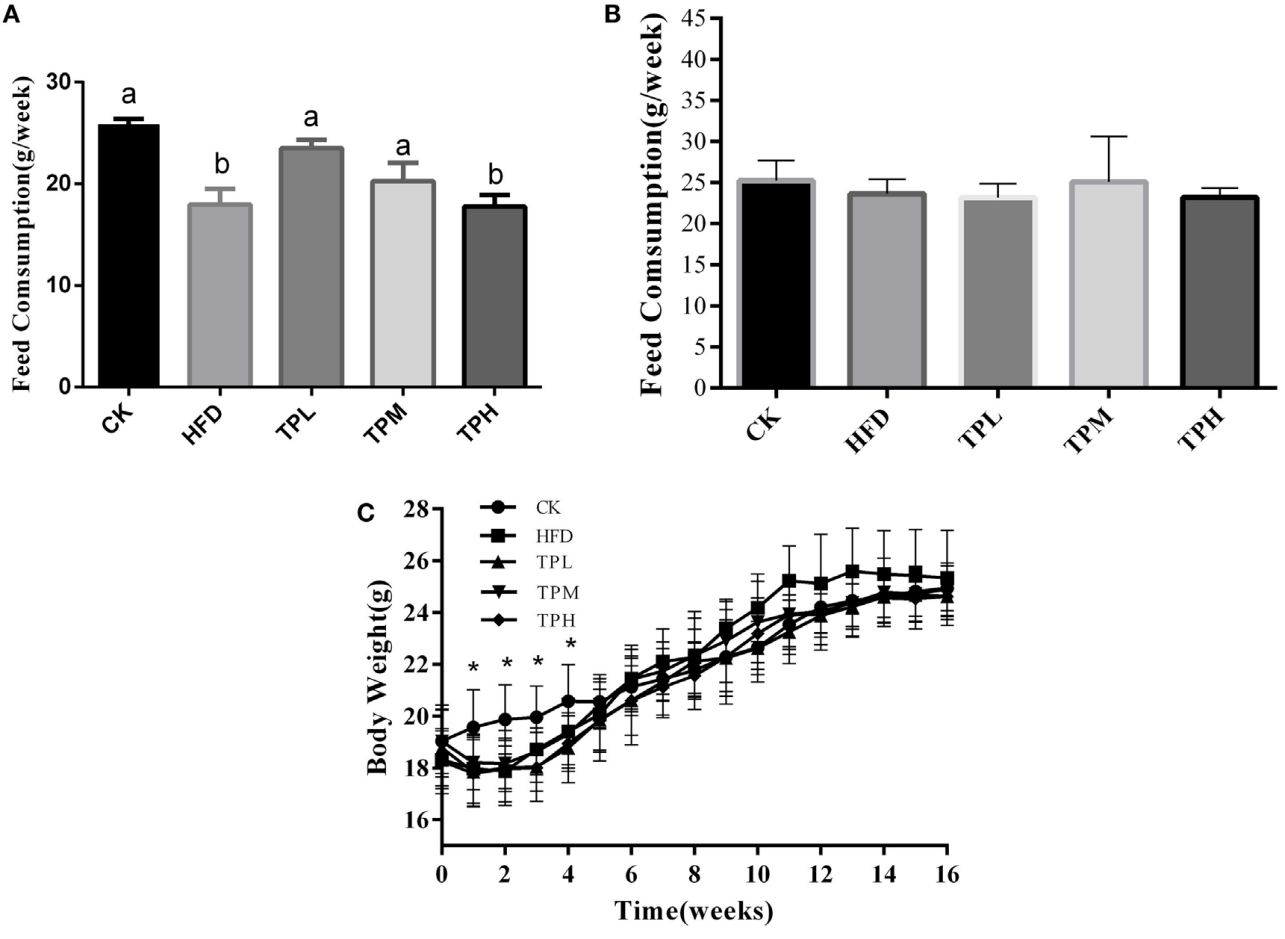
**Effects of diet with tea polyphenol on feed consumption and body weight of C57BL/6 ApoE^−/−^ mice**. **(A)** Feed consumption in 1st week; **(B)** feed consumption in the whole period; **(C)** body weight per mouse. Bars represent means ± SEM, *n* = 10. (a,b) Different lowercase letters indicate significant differences (*p* < 0.05); the same lowercase letter indicates that the difference is not significant (*p* > 0.05); *means significant differences compared with CK group (*p* < 0.05).

ApoE^−/−^ mice mean consumption of high-fat feed was less than that of the CK group, and the HFD and TPH groups had significantly lower food consumption (*P* < 0.05) in the first week (Figure 1A). However, the difference in feed consumption per week among TPH, TPM, TPL, HFD, and CK groups were not statistically different from the second week and over the whole study (*P* > 0.05) (Figure 1B). Figure 1C shows that the mean body weight of the TP group (TPH, TPM, and TPL) and HFD group were significantly lower than the CK group (*P* < 0.05) from the first week to the fourth week. The ApoE^−/−^ mice in the HFD group gained weight faster than the other groups from 5th week and had the highest weight from the 11th week to the end of the study. However, this difference was not significant (*P* > 0.05).

### TC, TG, HDL-C, and LDL-C of ApoE^−/−^ Mice Serum

**Figure 2:**
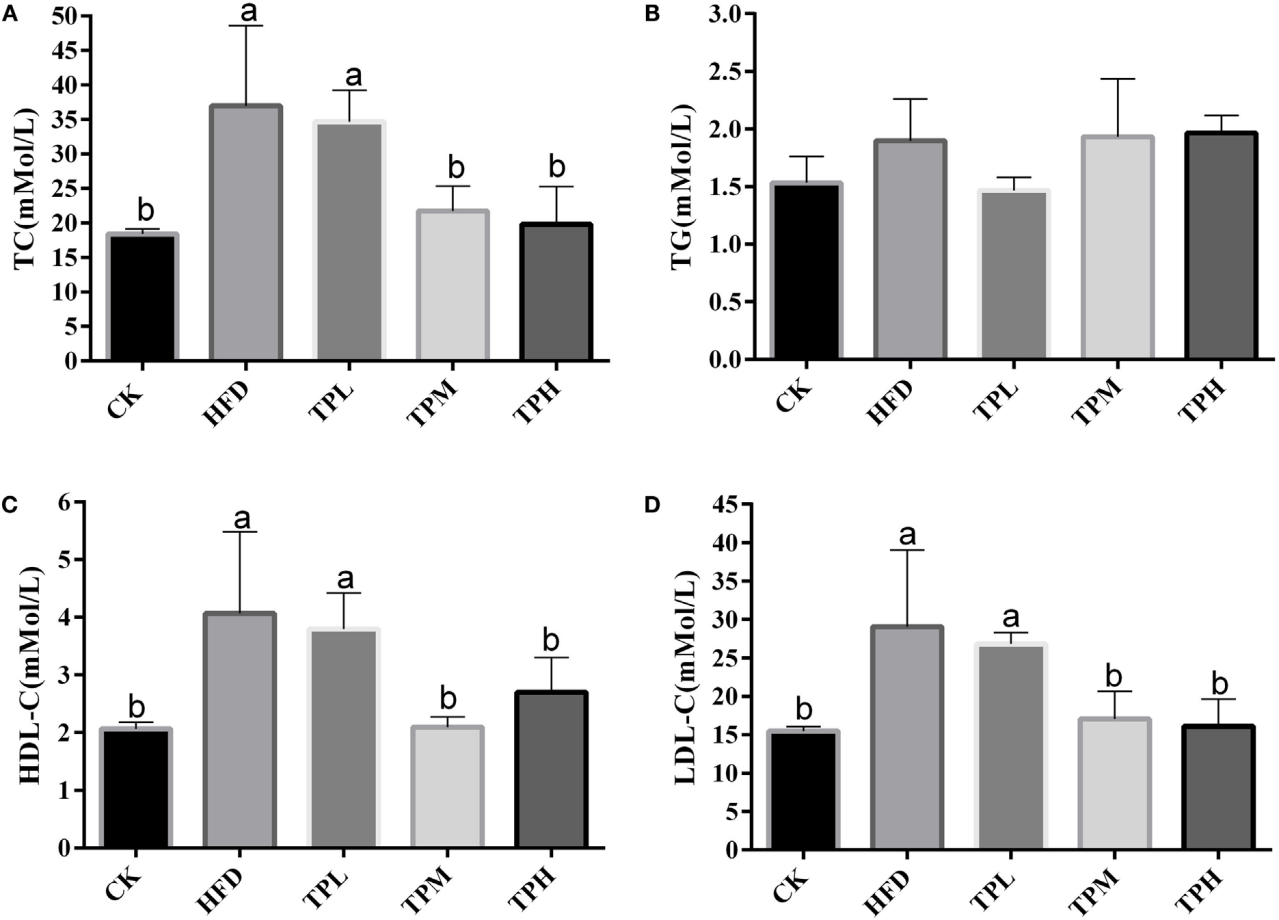
**Effect on the serum levels in the C57BL/6 ApoE^−/−^ mice after diet with tea polyphenol 16 weeks**. **(A)** TC; **(B)** TG; **(C)** LDL-C; and **(D)** HDL-C. Bars represent means ± SEM, *n* = 3. (a,b) Different lowercase letters indicate significant differences (*p* < 0.05); the same lowercase letter indicates that the difference is not significant (*p* > 0.05).

The effect of treatments on lipid parameters is demonstrated in Figure 2. After a HFD for 16 weeks, the mean serum TC (36.4 ± 12.2 mmol/L), LDL-C (28.4 ± 10.8 mmol/L), and HDL-C (4.1 ± 1.4 mmol/L) levels in the HFD group were significantly higher than those in the CK group (18.4 ± 0.7, 15.5 ± 0.6, and 2.1 ± 0.1 mmol/L, respectively) (*P* < 0.05).

No significant difference in serum TG level was found between the HFD and CK group. In the TPL group, no significant difference in lipid parameters was observed compared to the HFD group. The serum TC, LDL-C, and HDL-C levels were significantly lower among the TPH and TPM groups, when comparing to the HFD group (*P* < 0.05).

However, TG levels in the TPH, TPM, and TPL groups were 2.0 ± 0.5, 1.9 ± 0.2, and 1.5 ± 0.1 mmol/L, respectively, with no significant differences among the TPH, TPM, TPL, and HFD groups (*P* > 0.05) (Figure 2B).

If the mice consume 0.25-mg TPs per gram body weight per day, it will get better protection on AS. These results are calculated from the following fact: each mouse consumes about 4-mL (contain 1.6 g/L TPs) water; each mouse’s average body weight is 25 g.

### Atherosclerosis Plaques in ApoE^−/−^Mice Abdominal Aortas

**Figure 3:**
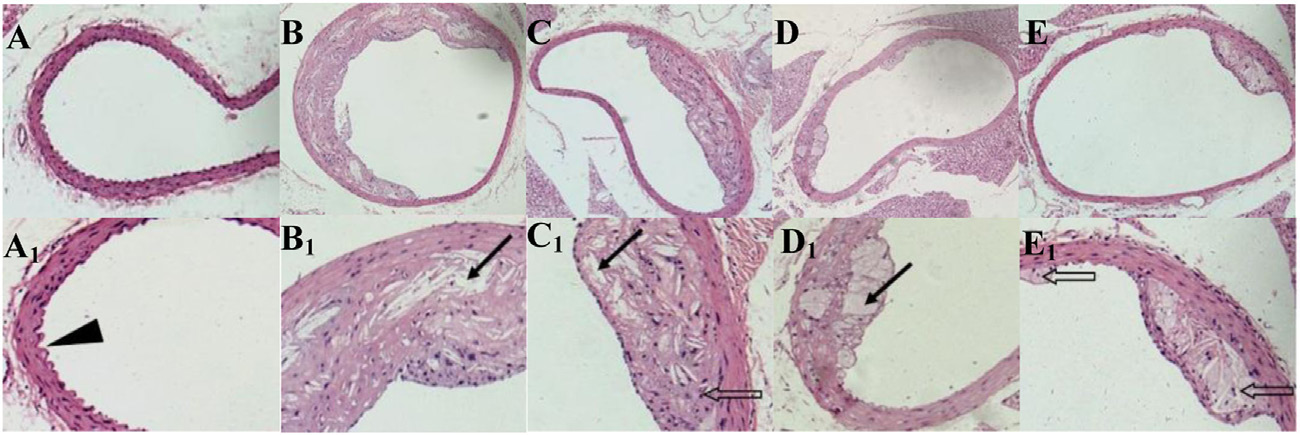
**Cross-sections of abdominal aorta stained by HE staining from C57BL/6 ApoE^−/−^ mice fed a normal diet (A), a high-fat diet (B) and tea polyphenols diet (C–E) for 16 weeks**. Representative photographs are also shown for abdominal aorta AS plaque area in C57BL/6 ApoE^−/−^ mice of CK group **(A,A1)**, HFD group **(B,B1)**, TPL group **(C,C1)**, TPM group **(D,D1)**, TPH group **(E,E1)**. Note: black triangle (►) is shown for intimal membrane protuberance; solid black arrows (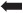) indicate AS material; and feint arrows (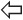) indicate foam cells.

**Figure 4:**
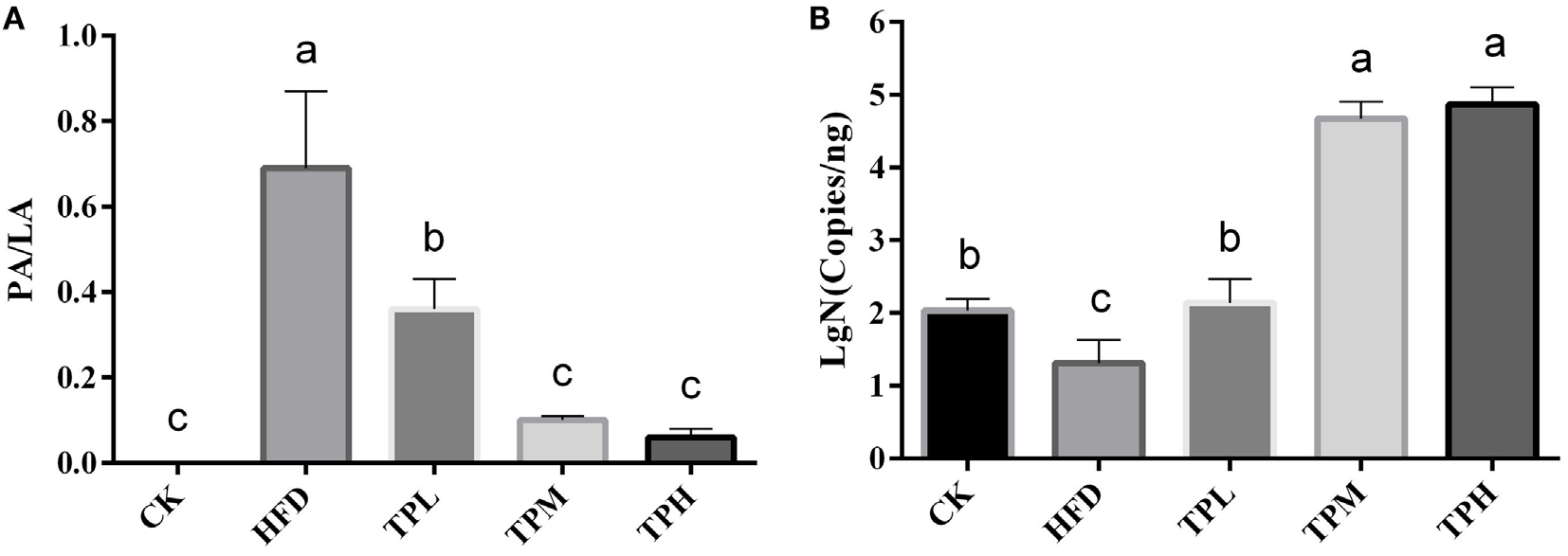
**Abdominal aorta AS plaque area and lumen area ratio (PA/LA, *P* < 0.01) in the C57BL/6 ApoE^−/−^ mice diet with tea polyphenol for 16 weeks (A)**. After a 16-week TP diet, *Bifidobacteria* copies in the TPL (*P* < 0.01), TPM (*P* < 0.001), and TPH (*P* < 0.001) groups were all significantly higher than that in the HFD (*P* < 0.01) group **(B)**. Bars represent means ± SEM, *n* = 10. (a,b,c) Different lowercase letters indicate significant differences (*p* < 0.05); the same lowercase letter indicates that the difference is not significant (*p* > 0.05).

ApoE^−/−^ mice abdominal aorta AS lesion progression was assessed by AS plaque size, intimal and medial integrity, and foam cell morphology observed by optical microscopy. The results indicated an association between the fat content in the feed and development of AS lesions: the PA/LA ratio in the HFD group was 0.69 ± 0.18, whereas no AS plaques were observed in the CK group. The ruptured foam cells formed AS plaques which accumulated in the intimal membrane, thus causing narrower blood vessels in mice of the HFD group. Therefore, the AS model in ApoE^−/−^ mice was successfully constructed with the HFD. After 16 weeks of treatment with TPs, microscopic analyses of the abdominal aorta lesions showed that atherosclerotic lesion development was retarded by dietary TP. AS plaque area in the abdominal aorta among TP fed groups were smaller than those in the HFD group (Figure 3). The PA/LA ratios in the TPL, TPM, and TPH groups were 0.36 ± 0.07, 0.10 ± 0.01, and 0.06 ± 0.02, respectively, and were significantly lower than those of the HFD group (0.69 ± 0.18) (*P* < 0.01) (Figure 4). No significant difference of AS lesion between the TPH and TPM groups (*P* > 0.05) was shown in this study; however, the AS lesion in TPH and TPM groups were significantly lower than that of the TPL group (*P* < 0.05).

### Copies of *Bifidobacteria* in the ApoE^−/−^Mouse Gut

The number of copies of *Bifidobacteria* was 1.31 ± 0.32 log copies/ng in feces of the HFD group, which was significantly lower than that in the CK group (2.03 ± 0.17 log copies/ng, *P* < 0.01). Therefore, a HFD may lower the amount of *Bifidobacteria* in the ApoE^−/−^ mouse gut. After a 16-week TP diet, *Bifidobacteria* copies in the TPL (2.46 ± 1.80 log copies/ng, *P* < 0.01), TPM (4.67 ± 0.23 log copies/ng, *P* < 0.001), and TPH (4.87 ± 0.23 log copies/ng, *P* < 0.001) groups were all significantly higher than that in the HFD group. Furthermore, copies in the TPM and TPH groups were markedly higher than those in the TPL group (*P* < 0.01), but there was no significant different between copy numbers in the TPH and TPM groups (*P* > 0.05). This illustrates that TP could increase copy numbers of *Bifidobacteria* in the ApoE^−/−^ mouse gut dose-dependently (Figure 4B).

### Non-Linear Dose–Response Analyses of TP, IB Copies, and AS Plaque

**Figure 5:**
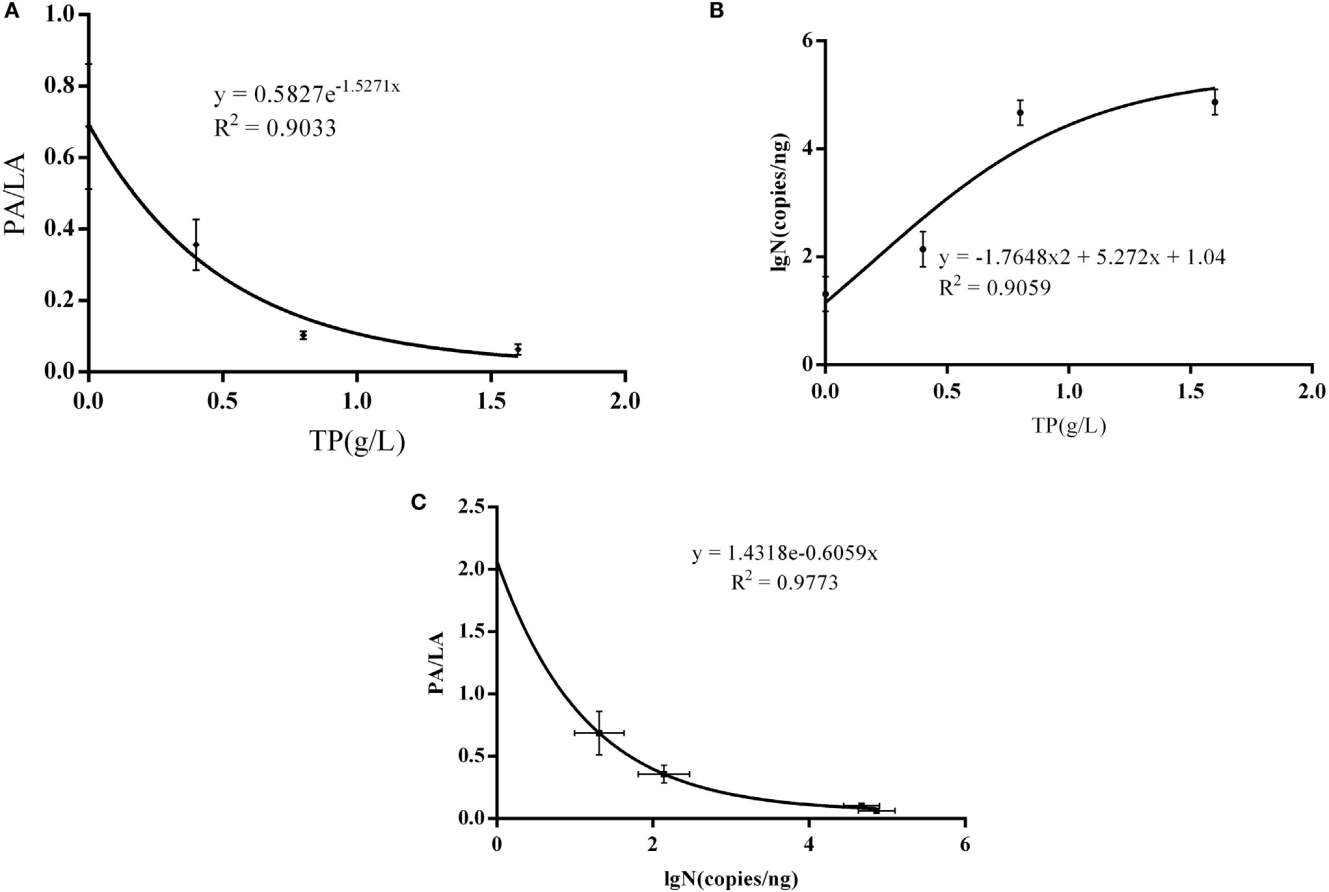
**Interaction between AS, IB, and TP**. **(A)** Correlations between TP doses and PA/PL ratio; **(B)** correlation between *Bifidobacteria* copies and PA/PL ratio; **(C)** correlation between TP doses and *Bifidobacteria* copies; the C57BL/6 ApoE^−/−^ mice fed a high-fat and tea polyphenols diet for 16 weeks. Bars represent means ± SEM, *n* = 3, **(A)** *P* = 0.0496, **(B)** *P* = 0.4839, and **(C)** *P* = 0.0114.

There was a significant non-linear dose–response association observed between AS plaque PA/PL value and the dose of TP (*R*^2^ = 0.9033, *P* = 0.0496) (Figure 5A), and it is also significant between PA/PL value and *Bifidobacteria* copy number (*R*^2^ = 0.9773, *P* = 0.0114) (Figure 5C); however, there no statistically significance between TP dose and the *Bifidobacteria* copy number (*R*^2^ = 0.9059, *P* = 0.4839) (Figure 5B).

## Discussion

Tea polyphenols, the most important bioactive ingredients present in tea, are believed to exert a diversity of beneficial effects in preventing the development of AS (32, 33). LDL-C is the most significant risk factor for AS development: LDL-C can be oxidized into ox-LDL, which may then interact with the scavenger receptors of macrophages and vascular smooth muscle membranes in the arterial wall. This interaction leads to the formation of fat-derived and muscle-derived foam cells; an AS plaque will develop due to the accumulation of the foam cell (34). It is believed that TPs may alleviate lipid metabolic abnormalities by reducing LDL-C levels (35) and inhibiting LDL-C oxidation (32, 33). The results of this study showed that TP could significantly retard AS lesion formation in ApoE^−/−^ mouse abdominal aorta. Moreover, the dose of TP was negatively correlated with the area of AS lesions.

Atherosclerosis is also considered as a type of chronic inflammation (36), which is closely related to metabolic diseases such as obesity and diabetes (36–38). In recent years, increasing evidence has found an association between metabolic disease and intestinal flora (39, 40). However, no study has fully demonstrated the relationship among the dietary polyphenols, the gut microbiota, and the development of AS. The further possible mechanism of TPs can inhabit AS development maybe the anti-inflammatory ability of itself.

Diet is the main environmental factor impacting the gut microbiome composition. We investigated the effects of a TP diet on ApoE^−/−^ mice fed a HFD, and our results showed that TP can dramatically increase the amount of gut *Bifidobacteria*. Lee (23) reported that growth of certain pathogenic bacteria, such as *Clostridium perfringens, Clostridium difficile*, and *Bacteroides* spp., was significantly repressed by TPs, while *Bifidobacterium* spp. and *Lactobacillus* spp. were less severely affected. According to the result that different intestinal bacteria had varying degrees of sensitivity to TPs, we concluded that TPs can increase the number of *Bifidobacteria*. It has been shown that *Bifidobacteria* species have beneficial effects on cardiovascular health. *B. breve* may significantly reduce the serum TC level when given to obese mice induced by HFD (14). In this study, we found that TP diet significantly reduced serum TC and LDL-C levels of ApoE^−/−^ mice (*P* < 0.05). Meanwhile, a recent metabolomic study indicated that product(s) yielded from microbial metabolism of lipids in the gut may promote AS (3).

It was believed that the serum HDL-C level is positively correlated with the existence of *Bifidobacteria* populations in the gut (5). However, this study found that TP diet significantly decreased (*P* < 0.05) the serum HDL-C levels in mice. A possible explanation is that TP diet decreases the serum cholesterol level, thus lowering the serum HDL-C level. HDL-C plays a key role in removing cholesterol from cholesterol-loaded macrophages, thus preventing the accumulation of arterial plaque (41, 42). When ApoE^−/−^ mice were fed with a HFD, the serum cholesterol level was elevated. This elevation induced the production of the HDL particles. Other studies have also found that a HFD may induce an elevation in serum HDL-C level (43), while a low-fat diet may decrease the serum HDL-C level (44).

Epidemiological studies have also showed that serum HDL-C level is inversely correlated with risk of cardiovascular events (41). However, our investigation demonstrated that the elevated serum HDL-C level in the HFD group did not play a protective role in preventing the formation of abdominal aorta AS plaques in ApoE^−/−^ mice (Figure 4A). It is important to note that HDL-C particles in ApoE^−/−^ mice are abnormal and disrupted (45) due to the lack of ApoE. The lack of ApoE in the HDL particles leads to a weaker cholesterol efflux capacity (the ability to remove cholesterol from cholesterol-loaded macrophages) (46–48). Hence, the formation of abdominal aorta AS plaques is expected. Furthermore, it is well known that AS is strongly associated with obesity. Zhao and his colleagues reported that *Bifidobacteria* may reduce fat accumulation (49). It was observed that the abundance of *Bifidobacteria* in the mouse gut declined significantly due to HFD feeding (50, 51). This phenomenon was also observed in our study (Figure 4B). A dose-dependent effect was found between the dietary TP and the number of *Bifidobacteria* in this study. The AS plaque PA/PL value was significantly and negatively correlated with the *Bifidobacteria* count. The dominant *Bifidobacteria* species in the ApoE^−/−^ mouse gut is *Bifidobacterium pseudolongum* (52). We make a putative opinion that TP could induce a *Bifidobacteria* boom, which could prevent AS development by modulating the fat metabolism. This process could be a part of the mechanism of the effect of TP on cardiovascular health.

## Author Contributions

The manuscript was written through contributions of all authors. All authors have given approval to the final version of the manuscript.

## Conflict of Interest Statement

The authors declare that the research was conducted in the absence of any commercial or financial relationships that could be construed as a potential conflict of interest.

## Abbreviations

AS, atherosclerosis; CK, normal diet group; HDL-C, high-density lipoprotein cholesterol; HFD, high-fat diet group; IB, intestinal *Bifidobacteria*; LDL-C, low-density lipoprotein-cholesterol; PA/LA, plaque area/lumen area; TC, total cholesterol; TG, triglycerides; TPH, tea polyphenols high-dose group; TPL, tea polyphenols low-dose group; TPM, tea polyphenols medium-dose group; TPs, tea polyphenols.
