# Supplementary material for: Impact of the Consumption of Tea Polyphenols on Early Atherosclerotic Lesion Formation and Intestinal Bifidobacteria in High-Fat-Fed ApoE−/− Mice
Source: Front Nutr. 2016 Dec 21;3:42. doi: 10.3389/fnut.2016.00042 (PMC5175490; doi:10.3389/fnut.2016.00042)
Supplement: Supplementary file 1 [file Table_1.PDF]

Table 1. Composition of the regular feed[a]

Ingredient	g/100g
Maize	34
Soybean Meal	15
Wheat Bran	15
Flour	15
Fish Meal	6
Ban Cake	3
Yeast Powder	2
Chicken Eggs	5
Canola Oil	1
Bone Meal	2.5
Sodium Chloride	2.5

[a]In addition,0.03% multivitamin was added based on the diet formula.
