# Supplementary material for: Impact of the Consumption of Tea Polyphenols on Early Atherosclerotic Lesion Formation and Intestinal Bifidobacteria in High-Fat-Fed ApoE−/− Mice
Source: Front Nutr. 2016 Dec 21;3:42. doi: 10.3389/fnut.2016.00042 (PMC5175490; doi:10.3389/fnut.2016.00042)
Supplement: Supplementary file 2 [file Table_2.PDF]

Table 2. Composition of Tea polyphenols[a]

	mg/g
Total Catechin	804
EGCG	455
EGC	175
ECG	89
EC	57
GCG	19
Caffeine	<0.1

[a]Means of triplicate analyses.
